# A biophysical framework for double-drugging kinases

**DOI:** 10.1073/pnas.2304611120

**Published:** 2023-08-17

**Authors:** Chansik Kim, Hannes Ludewig, Adelajda Hadzipasic, Steffen Kutter, Vy Nguyen, Dorothee Kern

**Affiliations:** ^a^Department of Biochemistry, Brandeis University, Waltham, MA 02454; ^b^HHMI, Brandeis University, Waltham, MA 02454

**Keywords:** kinase, conformational equilibrium, cooperativity, double-drugging

## Abstract

Selective orthosteric inhibition of kinases has been challenging due to the conserved active site architecture of kinases and emergence of resistance mutants. Simultaneous inhibition of distant orthosteric and allosteric sites, which we refer to as “double-drugging”, has recently been shown to be effective in overcoming drug resistance. However, detailed biophysical characterization of the cooperative nature between orthosteric and allosteric modulators has not been undertaken. Here, we provide a quantitative framework for double-drugging of kinases employing isothermal titration calorimetry, Förster resonance energy transfer, coupled-enzyme assays, and X-ray crystallography. We discern positive and negative cooperativity for Aurora A kinase (AurA) and Abelson kinase (Abl) with different combinations of orthosteric and allosteric modulators. We find that a conformational equilibrium shift is the main principle governing cooperativity. Notably, for both kinases, we find a synergistic decrease of the required orthosteric and allosteric drug dosages when used in combination to inhibit kinase activities to clinically relevant inhibition levels. X-ray crystal structures of the double-drugged kinase complexes reveal the molecular principles underlying the cooperative nature of double-drugging AurA and Abl with orthosteric and allosteric inhibitors. Finally, we observe a fully closed conformation of Abl when bound to a pair of positively cooperative orthosteric and allosteric modulators, shedding light on the puzzling abnormality of previously solved closed Abl structures. Collectively, our data provide mechanistic and structural insights into rational design and evaluation of double-drugging strategies.

## Significance

While immensely successful, drugging kinases by active site inhibitors has faced major challenges. Selectivity issues leading to side effects and emergence of resistance mutations rendered treatments targeting active sites ineffective. Double-drugging via active and allosteric sites is a recently developed approach to overcome these obstacles. Using Aurora A and Abelson kinase, we provide a quantitative biophysical evaluation of double-drugging by rationally selecting inhibitor combinations with positive cooperativity. The results shed light on the interplay of kinase conformational equilibria and inhibitor-dose requirements for effective inhibition. Due to our rational selection of a positively cooperative drug combination for Abl, we deliver a fully closed, inactive Abl structure, including regulatory SH3 and SH2 domains. Collectively, this biophysical framework aids future rational double-drug designs.

Protein allostery is one of the fundamental regulatory mechanisms involved in various biological processes (1). Specifically, the allosteric regulation of protein kinases has been found essential for signaling cascades. Thus, dysregulation and overexpression of protein kinases are often related to many human diseases, including various cancers. However, due to the highly conserved catalytic site architecture of kinases, specific orthosteric inhibition is often unsuccessful, causing off-target effects (2). In addition, cancers often develop resistant mutations circumventing treatments with orthosteric drugs (3, 4). To overcome these problems, the field has been exploring allosteric sites of kinases for specific and efficacious inhibition (5, 6).

A recently approved allosteric inhibitor of Abelson kinase (Abl), asciminib, has been highly effective in inhibiting Abl in vitro and in vivo (7–12). Remarkably, dual inhibition of Abl with this allosteric inhibitor combined with the orthosteric inhibitors (including imatinib, nilotinib, and ponatinib), which we refer to as “double-drugging”, has been impressively successful in abolishing the emergence of resistant mutants for Abl (12–14). Considering this clinical benefit, this approach has been applied to inhibit other targets such as EGFR kinase and SHP2 phosphatase (15, 16). However, the biophysical mechanisms underlying double-drugging of distant orthosteric and allosteric sites have not been well studied.

Herein, we provide the quantitative framework for double-drugging using two targets: Aurora A kinase (AurA) and Abl. Both kinases participate in various cellular pathways, and their dysregulation results in a multitude of cancers, such as breast cancer and leukemia (17–19). Common obstacles faced by orthosteric inhibitors for AurA and Abl include cytotoxicity, off-target effects, and emergence of resistance mutants (3, 4, 20, 21). For both systems, we exploit a rational selection of ligands to probe positive and negative cooperativity between remote orthosteric and allosteric sites using isothermal titration calorimetry (ITC), Förster resonance energy transfer (FRET), and coupled-enzyme assays. We find that both orthosteric and allosteric ligands exhibit preferred binding to the active or inactive states and that cooperativity occurs by shifting this active–inactive conformational equilibrium through long-range allosteric networks that are encoded for natural regulation of those kinases. X-ray crystal structures of the double-drugged complexes shed light on the atomistic mechanisms of cooperativity. After we determine negative cooperativity for the double-drug combination used by Novartis in their clinical trials, we rationally chose a different orthosteric inhibitor, Src inhibitor 1 (SKI), for positive cooperativity with asciminib. This double-drug combination forms a unique ternary complex, revealing a fully closed Abl structure.

## Results

### Cooperative Binding between Orthosteric and Allosteric Modulators of AurA.

**Fig. 1.**
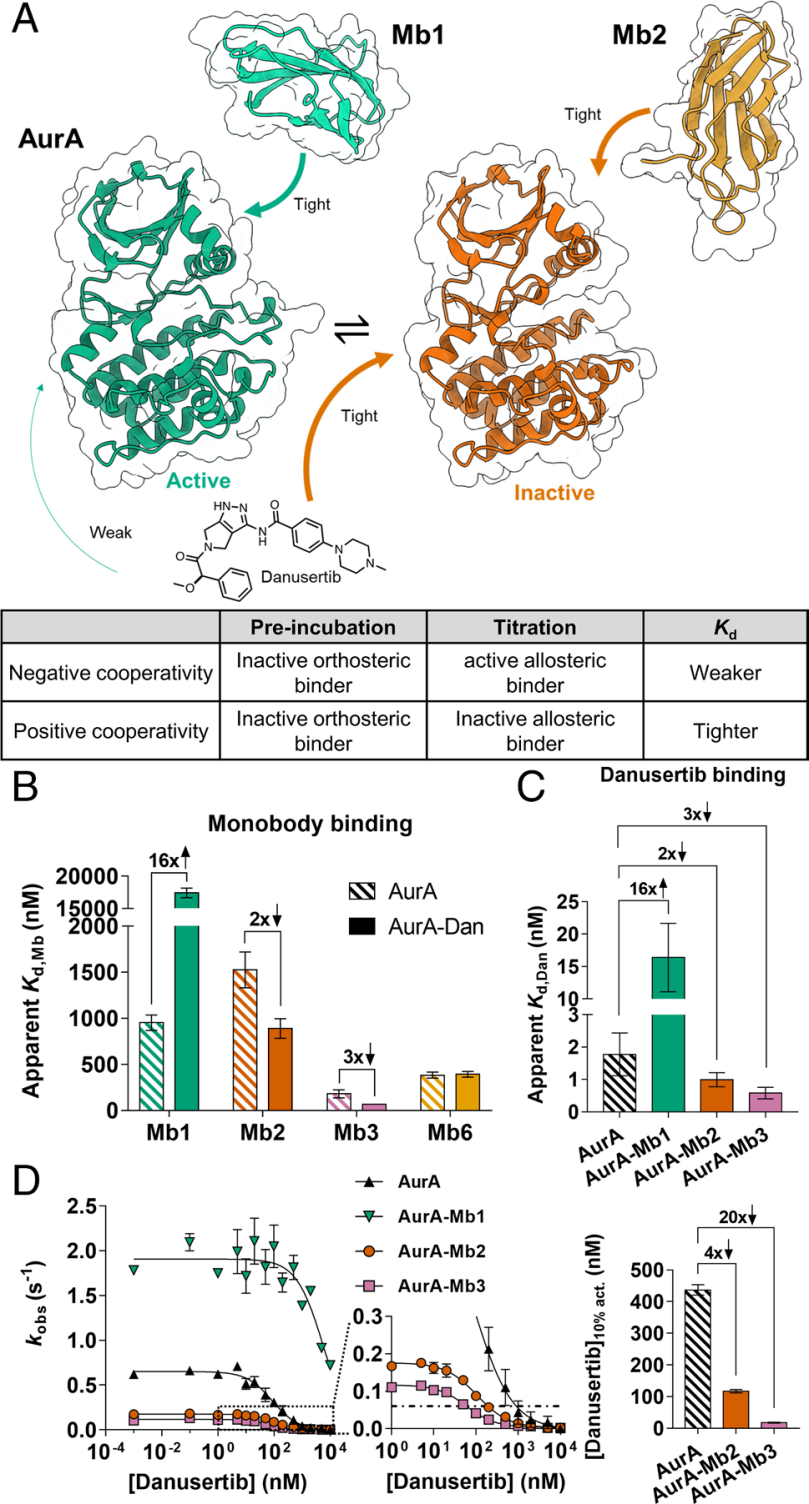
Double-drugging of AurA kinase with orthosteric drug danusertib and different allosteric modulators. (*A*) Schematic representation of active/inactive equilibrium of AurA [green, PDB-ID: 5G15, and orange, PDB-ID: 6C83 (25)]. Arrows indicate binding of danusertib and Mbs to their preferred AurA conformations. The table represents the rationale of positive and negative cooperativity for double-drugging of AurA. (*B*) Effect of preincubation with danusertib on observed dissociation constants (apparent *K*_d_) of different monobodies measured by ITC. Activating monobody Mb1 shows 16-fold negative cooperativity, while inhibiting monobodies, Mb2 and Mb3, show twofold and threefold positive cooperativity, respectively. Mb6 binding is not affected by the presence of danusertib (*SI Appendix,* Fig. S3). (*C*) Reversal of preincubation order during affinity measurements shows identical cooperativities for orthosteric/allosteric ligand combinations (*SI Appendix,* Fig. S1). Errors in (*B* and *C*) ITC data bar graph represent 68.3% CI (±1 SD) of the fit of the data. (*D*) Kinase inhibition curves of AurA and AurA in the presence of saturating concentrations of Mb1, Mb2 and Mb3 as a function of danusertib concentration. Enzyme assays were conducted (*n* = 2, mean ± SDM) under *k*_cat_/*K*_m_ condition with 3 mM Lats2 peptide, measuring observed activity (*k*_obs_). With inhibiting Mb2 and Mb3, fourfold and 20-fold lower concentrations of danusertib, respectively, are required to inhibit to 10% residual AurA activity [(Danusertib)_10% act._, and dashed line]. Errors in this bar graph were determined by jackknifing the inhibition curve data.

In solution, AurA exists in a conformational equilibrium between active and inactive states (22–24). We previously designed monobodies (Mbs) that are fully selective allosteric modulators, which bind to the natural allosteric regulatory site of AurA on the N-terminal lobe (N-lobe), the binding site for the natural coactivator protein TPX2 (25, 26). Different monobodies either act as activators or inhibitors depending on how they shift the active/inactive conformational equilibrium of AurA (25). To achieve double-drugging on AurA, we combined these Mbs with the orthosteric inhibitor danusertib (PHA739358) that tightly binds to AurA [IC_50_ = 13 nM, *K*_i_ = 0.87 ± 1.44 nM] (27). Since it has been shown that danusertib preferentially binds to the inactive conformation of AurA (22, 28), we hypothesized that inhibiting Mbs would bind tighter to AurA when in complex with the orthosteric inhibitor danusertib. Conversely, binding of activating Mbs to AurA should be weakened in the presence of danusertib (Fig. 1*A*).

Aligning with our hypothesis, we find that the binding affinity of activating monobody (Mb1) to AurA weakens 16-fold when AurA is presaturated with danusertib (Fig. 1*B*). To test whether the binding of Mb1 and danusertib is mutually exclusive, we repeated this experiment by preincubating AurA with a higher concentration of danusertib (*SI Appendix*, Fig. S1*E*). Identical Mb1 binding affinities, independent of saturating danusertib concentrations, reveal that the simultaneous binding of Mb1 and danusertib to AurA is possible. Thus, we reason that this 16-fold negative cooperativity for Mb1 binding arises from a conformational equilibrium shift of AurA to the inactive state induced by danusertib.

To achieve desired positive cooperativity between allosteric and orthosteric binders to AurA, we chose the inhibiting monobodies Mb2 and Mb3 because i) Mb2 is an inhibiting monobody for which we had obtained an X-ray crystal structure in complex with AurA, ii) Mb3 exhibits larger inhibition than Mb2, and iii) AurA-Mb3 complex exists in a monomeric form unlike the dimeric AurA-Mb2 complex (25). We indeed measure a twofold tighter binding of Mb2 to the AurA-danusertib complex compared to apo AurA (Fig. 1*B*). Using the equilibrium constant for active/inactive states of AurA previously determined [*K*_eq_= 0.67 (22)] and assuming identical affinities of Mb2 to the inactive states of apo- or danusertib-bound AurA, we fit our apparent affinities to a reversible two-state allosteric model. We find that the twofold positive cooperativity can be explained solely by the shift in the conformational equilibrium (*SI Appendix*, Fig. S2). Thus, a further increase in positive cooperativity would only be possible if the Mb affinity was tighter to the inactive state of the AurA-danusertib complex than to the inactive state of apo AurA. We indeed observed a threefold positive cooperativity for Mb3 with danusertib (Fig. 1*B*). We speculate that this increased affinity of Mb3 to the AurA-danusertib complex compared to apo AurA could result from favorable interactions with a closed activation loop, since danusertib binding shifts the equilibrium of the activation loop toward such conformation (23, 28).

To confirm whether the mechanism of cooperativity between Mbs and danusertib follows a classic allosteric model, we tested binding of Mb6 to the AurA-danusertib complex. Despite high affinity, Mb6 binding does not change AurA’s activity, implying that Mb6 binding does not shift the active/inactive conformational equilibrium of AurA (25). Indeed, the binding affinity of Mb6 to AurA is not changed in the presence of danusertib (Fig. 1*B* and *SI Appendix*, Fig. S3).

For a reversible two-state allosteric model, the same fold-change of cooperativity must be observed when reversing the order of binding. To measure changes in the affinity of danusertib upon Mb binding, we had to employ competitive replacement ITC with adenosine 5′-(α, β-methylene) diphosphate (AMPCP), since danusertib binds too tightly to AurA for direct measurement. Indeed, the measured cooperativities are matching quantitatively regardless of the binding order (Fig. 1*C*).

### Double-Drugging of AurA Lowers Inhibitor Concentration Needed for Efficacious Inhibition.

Next, we probed biological relevance of these observed cooperativities by measuring the inhibition of AurA kinase activity using Lats2 peptide as a substrate with cellular ATP concentrations. Preincubation of inhibiting Mbs resulted in a vastly decreased amount of danusertib required to cause 90% inhibition of AurA activity (Fig. 1*D*). This combined inhibition effect is the direct consequence of positive cooperativity. For instance, Mb3, which displays a larger degree of positive cooperativity than Mb2, causes a larger reduction in required amount of danusertib for effective inhibition.

### X-Ray Crystal Structures of Ternary Complexes: AurA-danusertib-Mb1 and AurA-danusertib-Mb2.

**Fig. 2.**
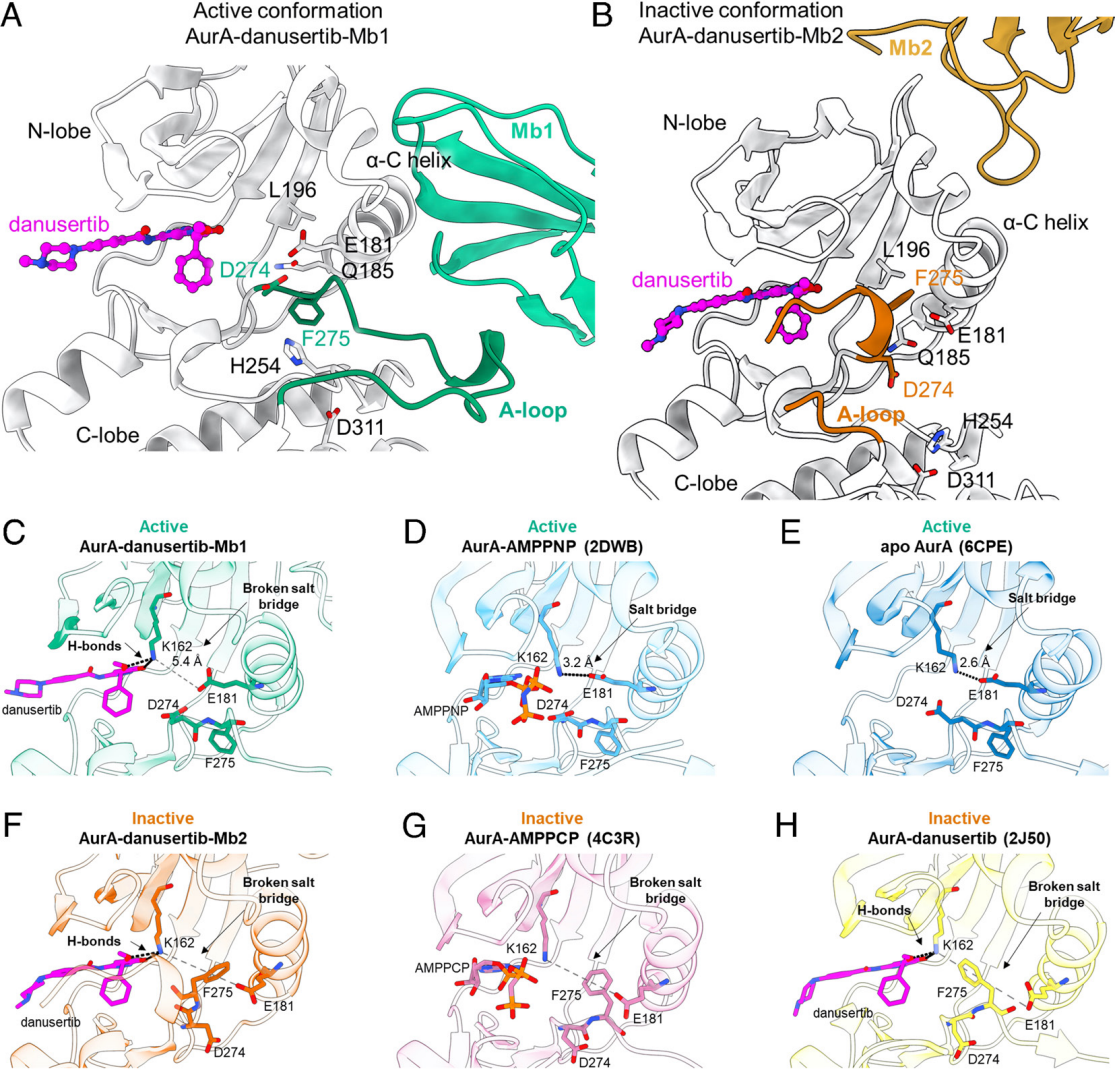
Proposed molecular mechanism for negative and positive cooperativity for double-drugging AurA with danusertib in combination with Mb1 and Mb2, respectively. (*A* and *B*) Zoom-in of X-ray crystal structures of AurA (gray) complexed with danusertib and either Mb1 (A, green) or Mb2 (B, gold). An intact regulatory spine, DFG-in conformation and extended activation loop in *A* is contrasted to a broken regulatory spine, DFG-out, and closed activation loop in (*B*). This closed activation loop provides additional hydrophobic interaction to the terminal ring of danusertib. (*C*–*H*) Orthosteric binding sites for six different AurA states reveal why danusertib has higher affinity for inactive AurA (22, 27, 31). K162 and E181, which form the canonical salt bridge in active AurA, and D274 and F275 (DFG-motif), are shown in stick representation. (*D*–*E*) While K162-E181 salt bridge is established in an active AurA conformations, (*C*) this salt bridge is broken in AurA-danusertib-Mb1 structure as K162 interacts with danusertib. (*F*–*H*) In the inactive AurA conformations, DFG-out F275 is positioned between K162 and E181, physically blocking the salt-bridge interaction, thereby prepositioning K162 for danusertib binding. (*A*–*H*) Oxygen, nitrogen, and phosphorous atoms are colored in red, blue, and orange, respectively. Carbon atoms are colored according to their respective protein cartoon.

We solved X-ray crystal structures of double-drugged AurA complexes to further understand the structural features responsible for the positive and negative cooperativity between Mbs and danusertib (*SI Appendix*, Table S1). The complex of AurA-danusertib-Mb1 [active, DFGin, BLAminus (29, 30)] displays hallmarks of an active kinase, such as an intact regulatory spine, the α-C helix in the “in” position, and the “DFG-in” state (Fig. 2*A*). However, we note that in contrast to the AurA-AMPPCP-Mb1 structure (PDB-ID: 5G15), D274 is rotated away from danusertib to avoid a steric clash with the terminal phenyl ring of danusertib (*SI Appendix*, Fig. S4*A*). This crystal structure corroborates the capability of danusertib to bind to the active conformation of AurA as we had tested biochemically (*SI Appendix*, Fig. S1*B*).

The most interesting structure for “double-drugging” with maximal inhibition is of course the ternary complex of AurA-danusertib-Mb2 [inactive, DFGinter (29, 30)]. Like AurA-AMPPCP-Mb2 (PDB-ID: 6C83), this ternary complex displays features of an inactive kinase: α-C helix “out”, “DFG-out,” as well as both a broken regulatory spine and a broken canonical salt bridge (K162-E181) (Fig. 2*B*). This is expected due to the conformational equilibrium shift caused by Mb2 binding and the preferential binding of danusertib to inactive AurA. Furthermore, the activation loop is fully shifted toward the active site, providing additional hydrophobic interactions to the terminal ring of danusertib (Fig. 2*B*). This shifted activation loop is a major structural feature of an inactive AurA (23, 28), as observed in AurA bound to the orthosteric inhibitor MLN8054 (PDB-ID: 2WTV) (32). The structure of AurA-AMPPCP-Mb2 (PDB-ID: 6C83) displays a similar activation loop, however, with an extended portion being disordered (residues 276 to 290) to circumvent clashing with the β- and γ-phosphate groups of AMPPCP (*SI Appendix*, Fig. S4*B*). The binary complex between AurA and danusertib (PDB-ID: 2J50) did not exhibit such a shift in the activation loop. However, it is unclear whether the activation loop conformation in the AurA-danusertib structure reflects the solution state since the activation loop is directly involved in crystal contacts (*SI Appendix*, Fig. S5).

Our crystal structures and ITC experiments showed that danusertib can bind to both the AurA-Mb2 and AurA-Mb1 complexes. To reveal why danusertib, however, binds with much higher affinity to AurA-Mb2 than AurA-Mb1 (there is no steric hindrance), we scrutinized the thermodynamic parameters of our ITC studies on danusertib binding to different AurA-Mb complexes (*SI Appendix*, Fig. S1 *A*–*D*). We find that the enthalpy for danusertib binding to AurA-Mb1 is reduced by 22.8 kJ/mol compared to AurA-Mb2, which approximates the equivalence of one salt bridge [12.6 to 20.9 kJ/mol (33)]. The canonical salt bridge between K162 and E181 is a feature of an active AurA, in both its apo form and bound to AMPPNP (PDB: 6CPE and 2DWB, respectively) (22) (Fig. 2 *D* and *E*). In contrast, the ternary complex of AurA-danusertib-Mb1 displays a broken salt bridge, as K162 interacts now with danusertib, while maintaining the α-C helix in the “in” position (Fig. 2*C*). Thus, we propose that the K162-E181 salt bridge in AurA-Mb1 must be broken for danusertib binding, as reflected by the lowered binding enthalpy. To confirm that apo AurA-Mb1 complex establishes the K162-E181 salt bridge, we deleted danusertib from the structure of AurA-danusertib-Mb1 and carried out molecular dynamics simulations in triplicate. We observed that K162-E181 indeed forms this salt bridge on average 80.8% in a 10 ns simulation (*SI Appendix*, Fig. S6*A*). However, in the presence of danusertib, we observe K162 to rather form a hydrogen bond with *O*-27 of danusertib’s methoxy moiety than with E181 in the MD simulation, which is the state sampled in our crystal structure as well (Fig. 2*C* and *SI Appendix*, Fig. S6 *B* and *C*).

In the inactive conformations of AurA, the broken K162-E181 salt bridge stems from the α-C helix and DFG-motif being positioned in the “out” conformation such that F275 positions between K162 and E181 (PDB: 4C3R and 2J50) (27, 31) (Fig. 2 *G* and *H*). Thus, we propose that K162 in inactive AurA conformations, such as AurA-Mb2 complex, is prepositioned for danusertib binding (Fig. 2*F*), which results in the tighter binding of danusertib to the inactive state of AurA.

### Cooperative Effect of Imatinib and Asciminib Binding on Abl.

**Fig. 3.**
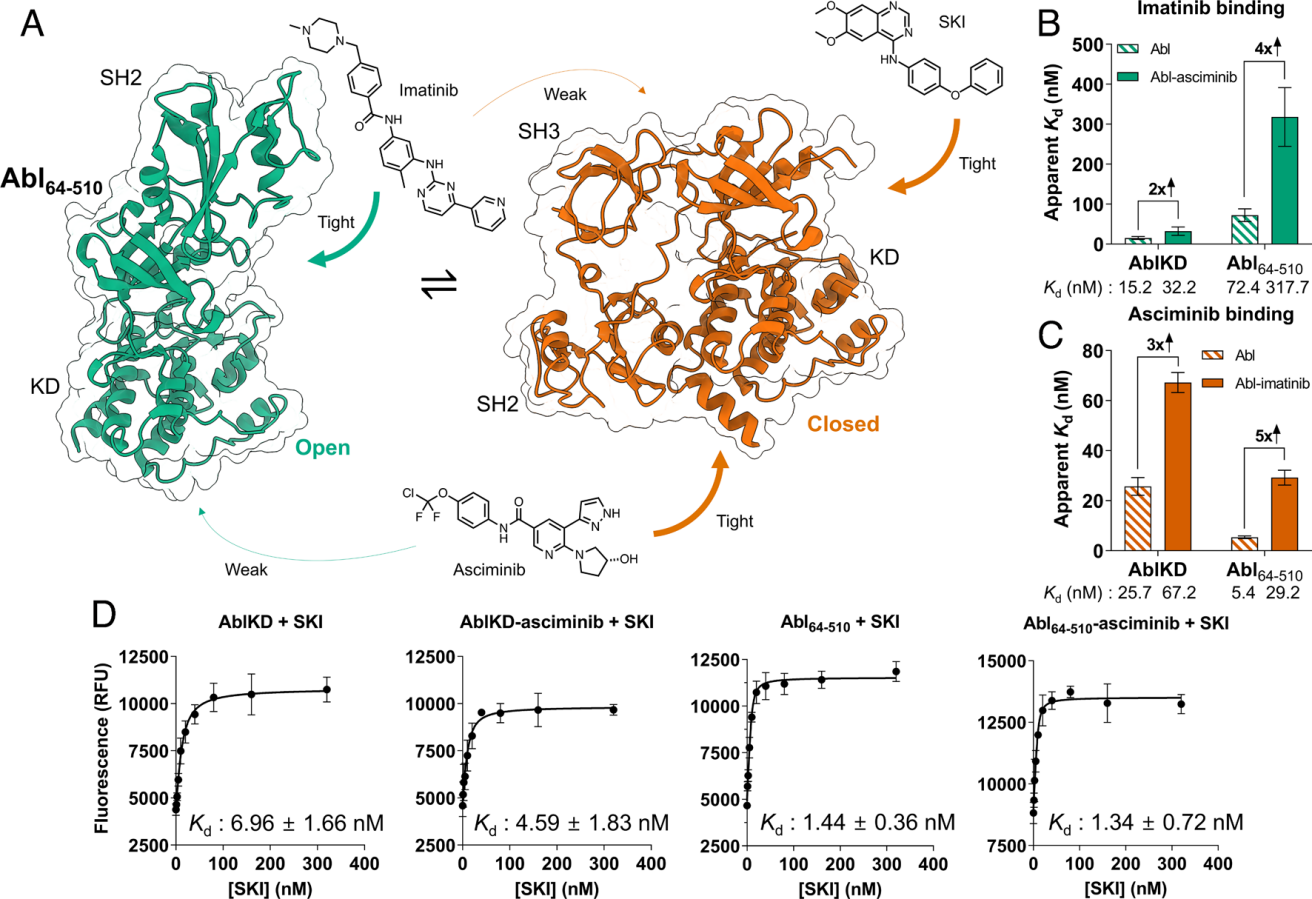
Double-drugging of Abl kinase. (*A*) Schematic representation of conformational equilibrium in Abl kinase. Arrows indicate binding of orthosteric inhibitors imatinib and SKI, and allosteric inhibitor asciminib to preferred Abl conformations. X-ray crystal structures from PDB-ID: 1OPL (green) and PDB-ID: 5MO4 (red) were used for open and closed Abl structures, respectively (12, 35). Note that the SH3 domain in the open structure is missing due to lacking electron density. (*B*) With ITC experiments, we observe twofold and fourfold negative cooperativity for open-conformation binder imatinib when AblKD and Abl_64–510_, respectively, are preincubated with closed-conformation binder asciminib (*SI Appendix,* Fig. S8). (*C*) Matching fold-change of negative cooperativity is observed reversing the order of modulators used in preincubation and titration, using ITC (*SI Appendix,* Fig. S8). (*B* and *C*) Errors in the ITC data bar graphs represent 68.3% CI (±1 SD) of the fit of the data. (*D*) FRET experiments to detect SKI binding (10 nM of enzyme in all experiments. Data (*n* = 2 to 5, mean ± SDM) have been fitted to quadratic binding equation. Unlike imatinib, SKI binds tighter to AblKD-asciminib and Abl_64–510_ than to AblKD. In AblKD, asciminib exhibits small positive cooperativity with the binding of SKI. *K*_d_ errors are SE of the fit.

Intrigued by our mechanistic insights into double-drugging of AurA, we turned to Abl, the only target currently in clinical trials for double-drugging. It has been shown that the combination of the orthosteric inhibitor imatinib and the allosteric inhibitor asciminib abolishes the emergence of resistance mutations (7–12), an impressive breakthrough. Therefore, Abl embodies a powerful target to delineate the biophysical constraints, or “framework”, for successful double-drugging. Since the quantitative biophysical parameters for this drug combination are not known, we set out to biophysically investigate the cooperativity and modulation of Abl’s open/closed conformational equilibrium first using this exact combination of orthosteric and allosteric inhibitors. Note that we use the well-established relevant construct of SH3-SH2-KD Abl (Abl_64–510_) (*SI Appendix*, Fig. S7). Abl exists in a conformational equilibrium between open, active, and closed, inactive conformations (34–36) (Fig. 3*A*). In the open conformation, the regulatory domains are elongated so that the SH2 domain moves onto the N-lobe of the kinase domain, forming a “top-hat” conformation (35). In the closed conformation, the regulatory domains tightly interact with the kinase domain, SH3:N-lobe and SH2:C-lobe, the latter facilitated by the bent C-terminal α-I helix (12, 35, 37) (Fig. 3*A*). This conformational equilibrium is susceptible to modulation by single agents such as imatinib and asciminib (12, 34, 38).

In ITC experiments, we find that imatinib binds fivefold tighter to AblKD, which exists exclusively in the open conformation, than to Abl_64–510_ (Fig. 3*B*). This confirms imatinib’s preferential binding to the open state of Abl (34). In full agreement with this model, imatinib binds to Abl_64–510_ with a fourfold decreased affinity in the presence of asciminib, since asciminib shifts the equilibrium to the closed state (12) (Fig. 3*B*). We conclude that this fourfold negative cooperativity between imatinib and asciminib stems from a shift in the conformational equilibrium of Abl, where both drugs preferentially bind to the open and closed conformation, respectively. Akin to AurA, preincubation of Abl_64–510_ with increased concentration of asciminib did not result in a weakened imatinib affinity, confirming the simultaneous binding of the two inhibitors (*SI Appendix*, Fig. S8*B*). Surprisingly, we found that imatinib and asciminib display a twofold negative cooperativity for AblKD (Fig. 3*B*). This implies the presence of an additional conformational equilibrium within the kinase domain itself (39) and that asciminib and imatinib shift this equilibrium in opposite directions. We refer herein to the asciminib-favoring conformation as the “closing-competent” conformation of AblKD.

Importantly, we measure identical negative cooperativities between imatinib and asciminib on both AblKD and Abl_64–510_, regardless of binding order, within the range of errors (Fig. 3*C* and *SI Appendix*, Fig. S8*E*). Due to the tight binding of asciminib, its affinity was measured via competitive replacement ITC using N-Myr peptide as a weak-binding ligand (12, 40). Collectively, we conclude that the binding of imatinib to the orthosteric site and asciminib to the allosteric site in Abl_64–510_ follow a two-state allosteric model, in which the two drugs favor the closed and open conformation, respectively.

### Positive Cooperativity between SKI and Asciminib on Abl.

Considering the negative cooperativity between imatinib and asciminib described by our ITC experiments, we wanted to rationally select an orthosteric inhibitor that exhibits positive cooperativity with asciminib. We chose Src inhibitor 1 (SKI), an orthosteric inhibitor that tightly binds to Src kinase (IC_50_ = 44 nM) (41, 42), because Bannister et al. recently measured that SKI preferentially binds to the α-C helix out, and thus closed-inactive conformation of Src kinase, despite the DFG-motif being in the “in” position (SKI was therefore traditionally classified as type I inhibitor) (Unpublished data, Bannister et al.). Due to Abl and Src kinases’ close structural homology, we hypothesized that SKI would bind to Abl in a similar fashion, thus exhibiting positive cooperativity with asciminib by preferentially binding to the closed state of Abl. Since SKI binding to Abl did not result in a detectable heat change in ITC, we turned to FRET experiments to quantify this interaction (Fig. 3*D* and *SI Appendix*, Fig. S9 and Fig. S10). SKI indeed binds preferentially to the closed conformation of Abl_64–510_, as seen by the fivefold tighter binding of SKI to Abl_64–510_ than to AblKD. Furthermore, we observe a modest positive cooperativity between SKI and asciminib binding in AblKD, indicating that SKI binds to the “closing-competent” conformation induced by asciminib. Unexpectedly, we did not find a difference between the binding affinities of SKI to Abl_64–510_ and Abl_64–510_-asciminib. We interpret this result as evidence that the conformational equilibrium of apo Abl is already far shifted to the closed conformation. Hence, the binding of asciminib had no effect on this equilibrium. This is, in fact, in agreement with a NMR study by Grzesiek and colleagues reporting overlapping chemical shifts between apo and GNF-5 (a predecessor of asciminib) bound Abl for open/closed equilibrium markers (34).

### Effect of Orthosteric and Allosteric Modulators on Abl Activity.

**Fig. 4.**
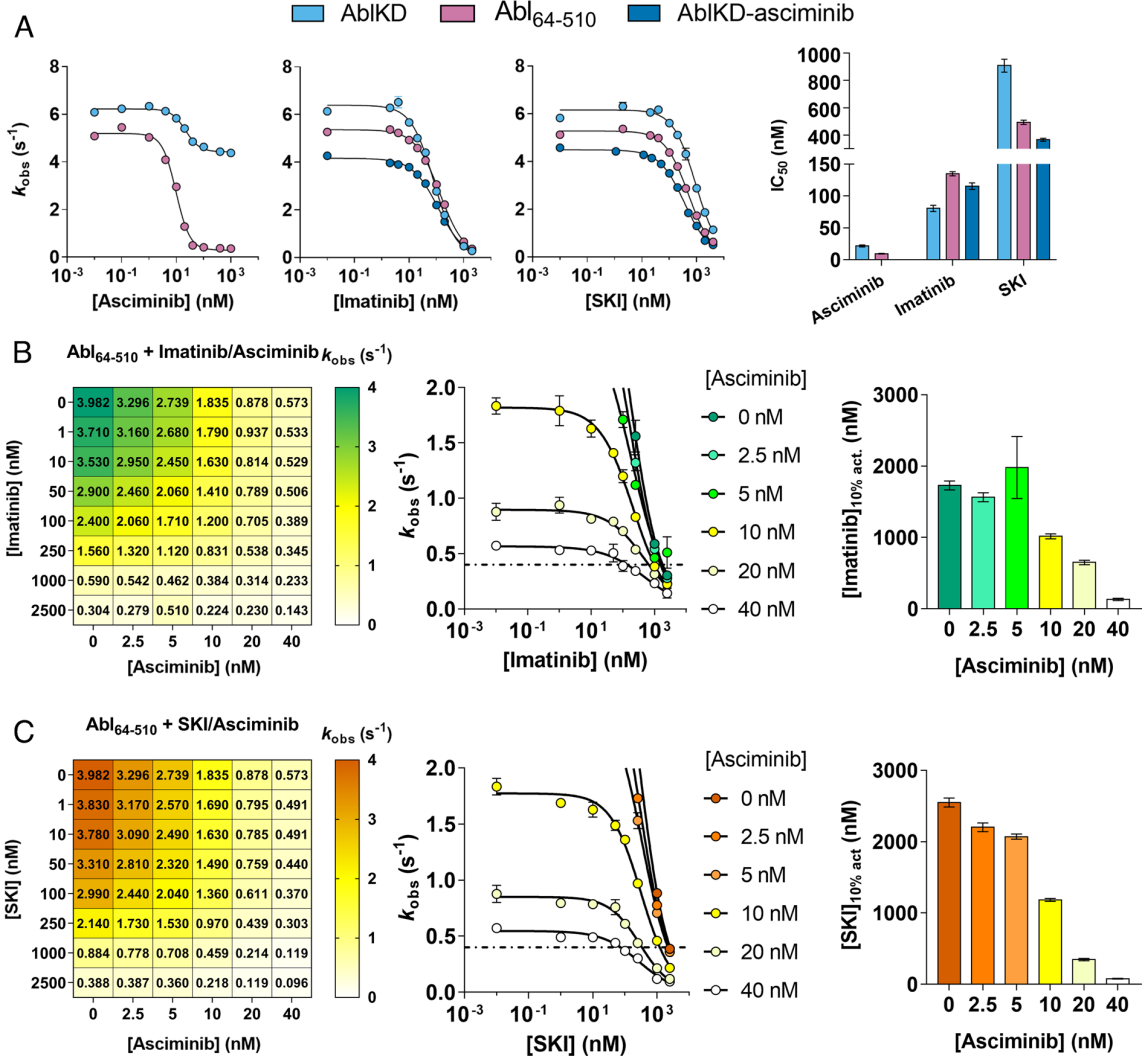
Catalytic activities of Abl kinase under double-drugging conditions. (*A*) Inhibition curves of AblKD and Abl_64–510_ with asciminib, imatinib, and SKI. IC_50_ values shift according to the favored binding conformations of the corresponding inhibitor. (*B* and *C*) In vitro synergy studies using (*B*) imatinib and asciminib (*C*) or SKI to examine cooperativity between both inhibitors for Abl_64–510_ activity. (*C*, *Left*) Numbers in the grid represent *k*_obs_ with respective concentrations of inhibitor combinations. (*C*, *Middle* and *Right*) graphic representation of the data to illustrate inhibitor concentration needed to achieve 10% residual kinase activity [(Imatinib)_10% act._ and (SKI)_10% act_, dashed line]. Note that SKI required for 10% residual kinase activity decreases with increasing asciminib, especially with higher fold-change than that observed for imatinib. All assays were measured (*n* = 4 for 0 nM orthosteric inhibitor, *n* = 2 for all other assays, mean ± SDM) under *k*_cat_/*K*_m_ condition with 2 mM Srctide. Errors in IC_50_ are SE of the fit. Errors in the bar graphs were determined by jackknifing the inhibition curve data.

Interestingly, it had been reported that allosteric inhibitors of Abl other than asciminib (such as GNF-2, GNF-5, myristate, and myristoyl-peptide) actually do not inhibit the catalytic activity despite binding to AblKD (40, 43, 44). However, with ITC, we observed that asciminib shifts the conformation of AblKD to the “closing-competent” conformation (Fig. 3 *B* and *C*). Is this “closing-competent” conformation of the kinase domain a catalytically inactive state of Abl? Inhibition curves of AblKD generated using a coupled-enzyme assay with Srctide as substrate and asciminib as an inhibitor reveal 30% inhibition at saturating asciminib concentration (Fig. 4*A*). We conclude that the closing-competent conformation of the kinase domain is indeed catalytically inactive and that asciminib shifts the conformational equilibrium of AblKD to be 30% in this conformation by binding to the C-lobe and allosteric propagation to the orthosteric site. This model also reconciles the moderate synergistic effect of SKI and asciminib binding to AblKD (Fig. 3*D*). We note that this unique allosteric propagation by asciminib could contribute to its increased potency relative to other myristate pocket binders. Most importantly, and stressing the importance of studying full-length kinases in drug development, asciminib causes a 93% inhibition of Abl_64–510_ at saturating concentration (Fig. 4*A*). This vastly increased inhibition is caused by the closing of the regulatory domains leading to an inactive kinase.

Next, we quantified the inhibition of AblKD and Abl_64–510_ by the two orthosteric inhibitors imatinib and SKI. In agreement with our affinity measurements (Fig. 3 *B–D*), imatinib exhibited a lower IC_50_ for AblKD than for Abl_64–510_, while SKI exhibited a higher IC_50_ for AblKD than for Abl_64–510_. Second, preincubation of AblKD with asciminib increased the IC_50_ for imatinib. This negative cooperativity arises from binding preferences of imatinib and asciminib to opposite conformations. In contrast, double-drugging of AblKD with SKI and asciminib resulted in a reduced IC_50_ since both have a binding preference to the same, “closing competent” conformation, highlighting their positive cooperativity (Fig. 4*A*).

We note that SKI’s IC_50_ is higher than imatinib’s IC_50_ with respect to AblKD, Abl_64–510_, and AblKD-asciminib (Fig. 4*A*), whereas this trend is reversed in our binding experiments (Fig. 3 *B*–*D*). We ascribe this discrepancy to the presence of ATP in the coupled-enzyme assay: AMPPCP binds twofold tighter to AblKD than Abl_64–510_ (*SI Appendix*, Fig. S11). Thus, under our assay condition, we reason that the ATP shifts the conformational equilibrium of Abl_64–510_ to the open state, which is favored by imatinib over SKI binding.

### Inhibition of Abl Kinase Activity under Double-Drugging Condition.

The key question for clinical application is: What is the effect of different dosing concentration combinations of the two inhibitors on Abl’s kinase activity? Therefore, we performed synergy studies on Abl_64–510_ kinase activity varying the concentration of both orthosteric and allosteric inhibitors (Fig. 4 *B* and *C*). These experiments underscore the negative cooperativity between imatinib and asciminib and corroborate the positive cooperativity between SKI and asciminib. First, we find a more pronounced inhibition of Abl_64–510_ by SKI than by imatinib in the presence of asciminib. On the other hand, when used as a single agent, imatinib inhibits Abl_64–510_ stronger than SKI, highlighting the difference in cooperativity. Second, we observe that in the presence of asciminib, less SKI is required for 90% inhibition of Abl activity compared to imatinib due to the positive cooperativity between SKI and asciminib (Fig. 4 *B* and *C*).

### X-Ray Crystal Structure of the Ternary Complex of Abl_64–510_-SKI-Asciminib.

**Fig. 5.**
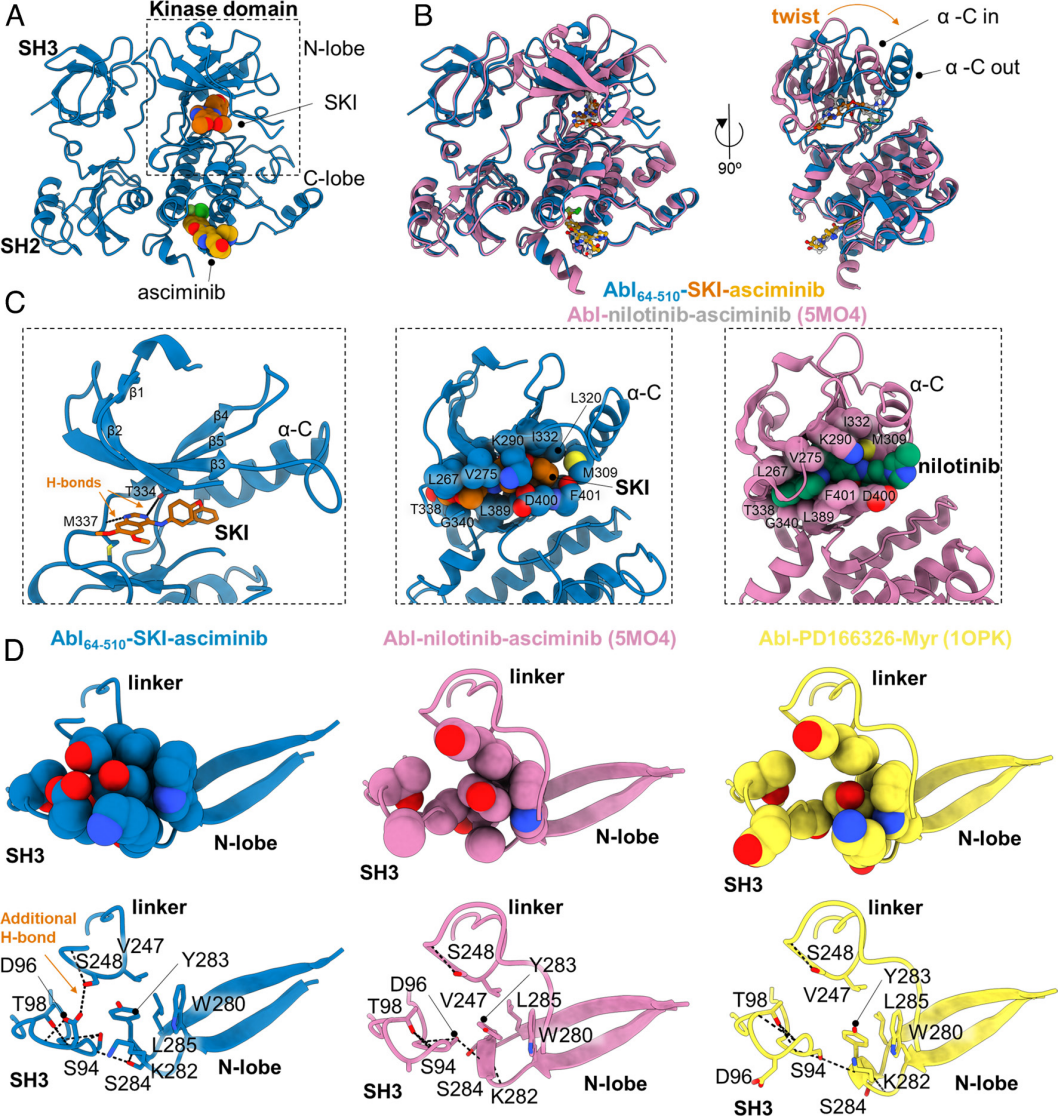
X-ray structure of ternary Abl_64–510_-SKI-asciminib complex reveals a fully closed conformation compared to previous “energetically frustrated” ternary closed Abl structures. (*A*) Abl_64–510_ bound to SKI and asciminib. (*B*) Superposition of Abl_64–510_-SKI-asciminib (blue) and Abl-nilotinib-asciminib (pink, PDB-ID: 5MO4) (12). When superimposed by the regulatory SH2 and SH3 domains, the N-lobe of Abl_64–510_-SKI-asciminib twists and exhibits α-C helix “out” position. (*C*) Zoom into the SKI and nilotinib binding sites. Van der Waals radii for the interacting Abl residues (spheres) with SKI (orange) and nilotinib (green) show more confined binding pocket for SKI than nilotinib. (*D*) Comparison of interface residues between N-SH3 domain (S94, D96, T98), linker (V247, S248), and N-lobe of kinase domain (W280, K282, Y283, S284, and L285) for Abl_64–510_-SKI-asciminib (blue), Abl-nilotinib-asciminib (pink, PDB-ID: 5MO4), and Abl-PD166326-myristate (yellow, PDB-ID: 1OPK) (12, 35). Due to the twist in the N-lobe for Abl_64–510_-SKI-asciminib, residues in the domain/domain interface exhibit better packing. For Abl_64–510_-SKI-asciminib, an additional hydrogen bond is established between S248 and D96 which contributes to this extended interface. Oxygen and nitrogen atoms are colored in red and blue, respectively. Carbon atoms are colored according to their respective protein cartoon.

Intrigued by the synergistic effect of SKI and asciminib on Abl activity, we structurally characterized this ternary complex by cocrystallization, resulting in a 2.86 Å crystal structure of Abl_64–510_-SKI-asciminib [inactive, DFGin, BLBplus (29, 30)] (Fig. 5*A* and *SI Appendix*, Fig. S12 and Table S1). Surprisingly, this Abl structure adopts a closed conformation with striking differences to previously reported closed structures; Abl in complex with nilotinib and asciminib (PDB-ID: 5MO4), as well as in complex with PD166326 and myristic acid, a groundbreaking structure of full-length Abl in the inhibited state (PDB-ID: 1OPK) (Fig. 5*B* and *SI Appendix*, Fig. S13) (12, 35). First, we note that the entire N-terminal lobe is ~30° twisted only for Abl_64–510_-SKI-asciminib, when aligned by the regulatory domains (Fig. 5*B* and *SI Appendix*, Fig. S13). Second, the α-C helix is adopting the “out” position resulting from this N-lobe twist, since an α-C helix “in position” would clash with strands β4 and β5 (Fig. 5 *B* and *C*). In consequence, the canonical salt bridge between K290 and E305, a hallmark of an active kinase, is broken in our structure, whereas D400 (DFG-motif) is positioned in the “in” position. Paradoxically, the two other closed ternary complexes of Abl possess an α-C helix located in the “in” position and an established canonical salt bridge (K290-E305), both reminiscent of an active kinase conformation (*SI Appendix*, Fig. S15), while their DFG-motif is in the “out” position. This highlights i) the importance of the α-C helix conformation and the canonical salt bridge, and not only the DFG-motif, in determining open/closed conformation which is directly correlated to active/inactive states in full-length kinases, and ii) that through binding of SKI and asciminib, we were able to capture the strictly closed and inactive conformation of Abl with regulatory domains. We note, that the orthosteric site is fully occupied by SKI and the twisted N-lobe aids in forming this tightly packed binding pocket (Fig. 5*C* and *SI Appendix*, Fig. S13). In fact, K290 located on β3 strand is wrapping over SKI burying the inhibitor in Abl’s orthosteric site. Besides extensive van der Waals interactions between SKI and Abl, the quinazoline ring of SKI shares two hydrogen bonds with Abl, one between the side chain hydroxyl of T334 on β-strand 5 and *N*-2 of SKI as well as between the amide of M337 and *N*-0 of SKI (Fig. 5*C*).

When compared to other closed Abl structures, we find an extended domain interface between SH3, linker, and N-lobe of the kinase domain, which explains the positive cooperativity between SKI and asciminib. This improved interface is a direct result of the twisted N-lobe. The repositioned β2 and β3 strands cause Y283 to be completely buried within this interface. In other ternary complexes of Abl, this interface is only partially formed (Fig. 5*D*). Moreover, S248 (located on the linker) forms a hydrogen bond with D96 in the SH3 domain, which is only present in the ternary complex of Abl_64–510_-SKI-asciminib. Strikingly, S248P was identified as a resistant mutation for GNF-2 and asciminib in cell culture-based screening (7, 45) with no mechanistic understanding, given that this mutation is far away from the allosteric inhibitor binding site. Our structure now reveals the important role of S248 in allosteric closing of Abl, and hence asciminib inhibition! We conclude that our complex of Abl_64–510_-SKI-asciminib represents the only example of a fully closed and inactive Abl structure.

## Discussion

To combat on-target cancer drug resistance, double-drugging holds promise to be a powerful strategy. The rationale behind is multiplication of individual resistance mutational probabilities for each drug. Impressively, combinations of asciminib and various orthosteric inhibitors, including imatinib, indeed abolish the emergence of Abl resistance mutants and this double-drugging of Abl is currently in clinical trials (12). Given these groundbreaking clinical results, we used Abl kinase to interrogate the biophysical mechanism underlying this drug combination to learn a quantitative biophysical framework for successful double-drugging. In a second step, we used our knowledge of conformational equilibria in kinases to rationally select alternative orthosteric drugs exhibiting improved synergy with the allosteric drug. Our results have major implications: i) Knowledge of conformational equilibria in drug targets indeed enables rational selection of inhibitor combinations with positive cooperativity and therefore better synergy. ii) Our Abl structure solves the apparent mystery of all previous closed Abl structures with the α-C helix in the active “in position” that contradicted the common features of inactive-closed kinases with the α-C helix in the canonical “out position”. Structural investigation of our double-drugged ternary complex with SKI and asciminib reveals a true α-C helix “out” state observed in Abl structures (*SI Appendix*, Fig. S14). This originates from an SKI-induced twist in the N-lobe causing a fully closed conformation, thus, releasing an energetically frustrated conformation observed in other double-drugged Abl complexes, since SKI and asciminib both preferentially bind to the closed state of Abl to cause positive cooperativity. In contrast, double-drugging with an open conformation binder (nilotinib) and a closed conformation binder (asciminib) results in an energetically frustrated Abl structure (PDB-ID: 5MO4). This structural study highlights how understanding of conformational equilibria crucially aids the discovery of further inhibited states. Our finding of the energetically frustrated conformation agrees with previous NMR experiments reporting an opposing binding preference of imatinib and GNF-5 for Abl (34). In contrast, Johnson et al. claimed that such an antagonism arises from mutually exclusive binding of orthosteric and allosteric inhibitors (38). This conclusion contradicts previous studies characterizing Abl-imatinib-GNF-5 by NMR as well as crystallographic studies on the ternary complex of Abl bound to both nilotinib and asciminib (12, 34). Our ITC studies resolve this controversy by ruling out mutual exclusivity for binding of imatinib and asciminib. iii) Our Abl data solve a heated debate: Recently, Kalodimos and colleagues argued that imatinib opens Abl via binding to its allosteric site (reported *K*_d_ >10 µM), and not via binding to its active site (46). This is in disagreement with NMR and cellular studies by Grzesiek et al. (34, 47, 48). Tighter binding of imatinib to open AblKD (*K*_d_ = 15 nM) compared to closed Abl_64–510_ (*K*_d_ = 72.4 nM) and negative cooperativity between imatinib and asciminib buttress Grzesiek’s model where imatinib’s preferential binding to the open conformation of Abl arises from its orthosteric site binding with nanomolar affinity.

Double-drugging has been applied to two additional targets, SHP2 phosphatase (16) and EGFR kinase (15, 49). Fodor et al. used a combination of two allosteric binders, SHP099 and SHP504, to inhibit the phosphatase SHP2 (16).The authors demonstrate that the combination reduces the dosage requirements of these allosteric inhibitors to achieve effective inhibition of SHP2; however, SHP504 is a very weak binder with an IC_50_ of 21 μM (16). For EGFR kinase, a combination of the inhibitor JBJ-04-125-02 binding right next to the irreversible orthosteric inhibitor osimertinib has been found to be more efficacious, than single agents, for inhibiting tumor growth in a mouse model. Furthermore, Jänne and colleagues demonstrated that this double-drugging resulted in the reduced emergence of resistance mutants in cellular assays (49). Here, the allosteric inhibitor binding site is in immediate proximity to the orthosteric site, resulting in direct interactions between the two inhibitors potentially driving positive cooperativity (15, 49).

In contrast, we investigated the mechanism of dual inhibition in AurA and Abl kinase targeting a distant allosteric site that is involved in natural regulation, in combination with active site drugs. Rationally targeting those natural allosteric sites has the advantage that it assures allosteric coupling to activity. We demonstrate with our amateur attempts on both kinases that rational selection of double-drug combinations with positive cooperativity, and hence increased synergy, is possible based on knowledge of involved conformational equilibria. Furthermore, we note that such kinase activity-based synergy studies could easily be performed in a high-throughput manner to test orthosteric and allosteric inhibitor combinations.

In summary, this work proposes a biophysical framework for designing and evaluating double-drugging synergy utilizing orthosteric and allosteric modulators. As highlighted here, positive cooperativity is desirable for double-drugging approaches improving selectivity and dosage requirements. However, while extreme negative cooperativity is undesirable, the clinical success of Novartis’ drug combination for Abl (12) with fourfold negative cooperativity as measured here suggests a clinical efficacy window ranging from small negative to strong positive cooperativity, given single-drug efficacy. Single drug efficacy is crucial, as otherwise a single resistance mutation abolishing binding of one drug would render the dual treatment to combat drug resistance essentially ineffective.

## Methods

### Cloning and Purification of Aurora A and Monobodies.

AurA (residues 122 to 403, TEV-cleavable, N-terminal His6-tagged, kanamycin-resistance) in pET28a and LPP (#79748) from Addgene were cotransformed in BL21(DE3) cells and plated on Kan/Spec LB plate. Expression cultures were grown in TB to OD = 0.6–0.8 and induced with 0.6 mM IPTG for 16 h at 21 °C. Harvested cells were resuspended in 50 mM Tris–HCl, 300 mM NaCl, 20 mM MgCl_2_, and 10% glycerol, pH 8.0, and sonicated in the presence of EDTA-free protease inhibitor cocktail, lysozyme and DNAse. Clarified lysate was purified via Ni-NTA columns. AurA was eluted in 100% of 50 mM Tris–HCl, 300 mM NaCl, 500 mM imidazole, 20 mM MgCl_2_, and 10% glycerol, pH 8.0, which was combined with TEV and GST-LPP, and then dialyzed overnight against 50 mM Tris–HCl, 300 mM NaCl, 1 mM MnCl_2_, 5 mM TCEP, and 10% glycerol, pH 7.5 at 4 °C. Cleaved Aurora A was purified with Ni-NTA and GST columns and subsequently polished with a 26/600 S200 pg gel filtration column equilibrated in 20 mM Tris–HCl, 200 mM NaCl, 20 mM MgCl_2_, 5 mM TCEP, and 10% glycerol, pH 7.5. Pure fractions were pooled and concentrated to around 40 μM, and stored in −80 °C. Monobodies (TEV-cleavable, N-terminal His6-tagged) were purified with on-column refolding as described in Zorba et al. (25).

### Cloning and Purification of AblKD and Abl_64–510_.

AblKD (residues 229 to 510, TEV-cleavable, N-terminal MBP-His6-tagged) and Abl_64–510_ (residues 64 to 510, TEV-cleavable, N-terminal MBP-His6-tagged) were cloned into pETm41 (GenScript) (*SI Appendix*, Fig. S7). Residue numbering follows Abl1b isoform that naturally consists of N-myristoylation. All Abl constructs were cotransformed with phosphatase YOPH (streptomycin-resistance) in BL21(DE3) cells and plated on Kan/Strep LB plate. Expression was performed in TB media and induced at OD = 0.6–0.8 with 0.1 mM (for AblKD) or 0.2 mM IPTG (for Abl_64–510_) for 16 to 20 h at 18 °C. Harvested cells were resuspended in 50 mM Tris–HCl, 500 mM NaCl, and 1 mM TCEP, pH 8.0 (buffer A). Cells were sonicated in the presence of EDTA-free protease inhibitor cocktail, lysozyme, and DNAse. Clarified lysate was with Ni-NTA columns. The protein was eluted with 100% 50 mM Tris–HCl, 500 mM NaCl, 500 mM imidazole, and 1 mM TCEP, pH 8.0 and combined with TEV and CIP (#M0525, NEB) and dialyzed overnight against buffer A at 4 °C. Cleaved Abl was further purified with Ni-NTA and a Q column (gradient elution with 50 mM Tris–HCl, 1 M NaCl, and 1 mM TCEP, pH 8.0). Prior to anion exchange chromatography Abl was dialyzed into 50 mM Tris–HCl, 1 mM TCEP, and 10% glycerol, pH 8.0. Dephosphorylated Abl fractions were polished with 26/600 S75 pg (for AblKD) or 26/600 S200 pg (for Abl_64–510_) gel filtration column with buffer A. Pure fractions were aliquoted to around 40 μM and stored in −80 °C.

### ITC.

All titrations were carried out using Nano ITC (TA Instruments) and analyzed via the NanoAnalyze software either using the independent fit model or competitive replacement model. The first injection of each experiment was discarded according to the software manual.

For AurA, danusertib (Selleckchem #S1107) was reconstituted to 100 mM in 100% DMSO and was diluted to appropriate concentration to match final 5% DMSO (vol/vol) for each experiment. An ADP-analogue, AMPCP, was used for competitive replacement experiments to measure and fitting of the binding of danusertib to AurA. All proteins were dialyzed in 20 mM Tris–HCl, 200 mM NaCl, 10% (vol/vol) glycerol, and 5 mM TCEP, pH 7.5. AMPCP was resuspended with the same buffer and was matched to pH 7.5. DMSO was added prior to each experiment to match 5% between titrant and titrand. Each injection was added in 2 µL increments with 180 s interval at a constant stirring speed of 300 rpm and at 25 °C. Concentrations used for the experiments are noted in *SI Appendix*.

For Abl, N-Myr peptide (Myr-GQQPGKVLGDQR), ordered from GenScript, was used for competitive replacement experiments to measure and fitting of the binding of asciminib to Abl. All proteins were dialyzed in 50 mM Tris–HCl, 500 mM NaCl, and 1 mM TCEP, pH 8.0. Imatinib-mesylate (Sigma #SML-1027) and asciminib (MedKoo #206490) were reconstituted to 10 mM in 100% DMSO and were diluted to appropriate concentration for each experiment. N-Myr peptide was resuspended with the same buffer and was matched to pH 8.0. DMSO was added prior to each experiment to match 5% between titrant and titrand. Each injection was added in 1 to 1.5 µL increments with 180 s interval at a constant stirring speed of 300 rpm and at 25 °C. Concentrations used for the experiments are noted in *SI Appendix*.

### In Vitro Kinase Assay.

To measure the IC_50_ of danusertib to AurA, ADP-Glo^TM^ Max assay (Promega #V7001) was used. 20 nM AurA in the absence or presence of either saturating concentration of Mb1 or Mb2 or Mb3 was incubated with 3 mM Lats2 (ATLARRD**S**LQKPGLE), 0.6 mg/mL BSA, and varying concentrations of danusertib with final 5% (vol/vol) of DMSO at 25 °C in 20 mM Tris–HCl, 200 mM NaCl, 10% (vol/vol) glycerol, and 5 mM TCEP, pH 7.50. The bolded and underlined residue indicates site of phosphorylation. The reaction was initiated by adding 5 mM ATP, and the final samples were collected after 2 h for AurA-Mb1 complex, 10 h for apo AurA, and 20 h for AurA-Mb2 and AurA-Mb3 complexes. The amount of ADP in the samples was measured by following the manufacturer’s protocol and used to calculate the observed rate.

Assays for Abl were performed at 25 °C with half-well 96-well plate (Corning #3994) in 50 mM Tris–HCl, 500 mM NaCl, and 1 mM TCEP, pH 8.0, supplemented with 20 nM Abl kinase (AblKD or Abl_64–510_), 2 mM Srctide (EI**Y**GEFKK), 0.6 mg/mL BSA, 20 mM MgCl_2_, 750 µM NADH, 6 mM PEP, and 2.5 units of PK/LDH (Sigma #P0294). The bolded and underlined residue indicates site of phosphorylation. Oxidation of NADH at A_340_ was monitored using SpectraMAX by starting the assay with 1 mM ATP. The final volume of the assay was 100 µL. The observed rate (*k*_obs_) was calculated following Zorba et al. (25).

All data were processed using GraphPad Prism and fitted to a four-parameter dose-response model.

### Molecular Dynamics Simulation.

All-atom molecular dynamics simulations were conducted using OpenMM 7.6 (50) and “Making it rain” cloud-based notebook environment (51). The structure of AurA-danusertib-Mb1 was used as an initial model. To mimic danusertib binding to AurA-Mb1 under ITC conditions, we created such structure via removal of danusertib from our ternary complex AurA-danusertib-Mb1 [since the published AurA-Mb1 structure (PDB-ID: 5G15) has AMPPCP bound to active site (25). Parameterization for all MD runs was conducted using LEaP (52) with Amber ff14SB force field (53), GAFF2 (54) for ligand, and TIP3P (55, 56) water model. The systems were neutralized with NaCl at 0.2 mM, following the ITC conditions, and box size was set at 20 Å. AurA-Mb1 and AurA-danusertib-Mb1 structures were equilibrated to 298 K via Langevin dynamics (57) and 1 bar via Monte Carlo barostat (58) with 2 fs integration time. We set 10,000 steps of energy minimization with 1,000 kJ/mol of harmonic position restraints. The systems were equilibrated for 0.2 ns and 1 ns for AurA-Mb1 and AurA-danusertib-Mb1, respectively, in the NVT ensemble. Then, with accordingly equilibrated systems, triplicates of 10 ns production runs were done in the NPT ensemble. Trajectories were analyzed using VMD 1.9.4a53 (59).

### FRET Measurements.

FluoroMax-4 (Horiba Scientific) with temperature controller (water bath) was used to measure FRET between intrinsic tryptophan fluorescence and SKI. Either 10 nM Abl or 10 nM Abl + 200 nM asciminib was preincubated with varying concentrations of SKI for 40 min at 25 °C before measurements. An increase in the fluorescence was measured when the complex, specifically tryptophan, was excited at 295 nm to emit at 340 nm, which then excites SKI to emit at 460 nm (*SI Appendix*, Fig. S9). Both 5 nm of excitation and emission slit width were used. Control experiments (buffer-only, protein-only, and inhibitor-only) were confirmed that the increase of fluorescence is caused by the fluorescence energy transfer.

The fluorescence intensity at 460 nm versus SKI concentration was fitted to the quadratic equation below in GraphPad Prism to obtain apparent *K*_d_.

$$\begin{array}{c} F=F_{0}+A\frac{\left(\left[I\right]+\left[E_{t}\right]+K_{d}\right)-\sqrt{{\left(\left[I\right]+\left[E_{t}\right]+K_{d}\right)}^{2}-4\;[E_{t}]\;[I]}}{2[E_{t}]} \end{array}$$

We simulated curves with tighter *K*_d_ for comparison to ensure that the fitted curves are not step functions due to the high enzyme concentration (*SI Appendix*, Fig. S10).

### Crystallographic Methods.

Crystals of AurA in complex with Mb1 and danusertib were obtained by combining 2 µL of 300 µM (10 mg/mL) AurA + 315 µM (4 mg/mL) Mb1 + 2 mM AMPPCP + 4 mM MgCl_2_ with 2 µL reservoir of 0.1 M MES pH 6.5 + 0.2 M ammonium sulfate + 4% (v/v) 1,3-propanediol + 15 to 18% PEG 8,000. Streak seeding was used to obtain bigger crystals. Crystals were grown at 18 °C by hanging drop. The crystals were transferred to a drop of fresh reservoir for 30 s to remove excess nucleotides from the crystal surface. Then, the crystals were transferred to a drop with reservoir with 1 mM danusertib for 16 h of soaking. For cryoprotection, the crystals were transferred into 17.5% PEG 400, 17.5% ethylene glycol, 15% reservoir, and 50% water for a few seconds.

Crystals of AurA in complex with Mb2 and danusertib were obtained by combining 0.5 µL of 300 µM (10 mg/mL) AurA + 315 µM (4 mg/mL) Mb2 + 1 mM danusertib with 0.5 µL of 0.1 M BIS–TRIS pH 5.5 + 0.2 M Ammonium acetate + 25% PEG3350. Crystals were grown at 18 °C by sitting drop. Crystals were harvested and subsequently flash frozen.

Diffraction data for AurA-danusertib-Mb1 and AurA-danusertib-Mb2 were collected at 100 K Advanced Light Source (Lawrence Berkeley National Laboratory) at beamlines BL821 and BL501, respectively, and were integrated with XIA2 (60) or XDS (61). Data were scaled and merged with AIMLESS (62). Initial phases were obtained with molecular replacement programs MOLREP (63) and PHASER (64) by using AurA + Mb1 + AMPPCP (PDB-ID: 5G15) for AurA-danusertib-Mb1 structure and AurA + AMPPCP (PDB-ID: 4C3R) and HA4Mb (PDB-ID: 3K2M) for AurA-danusertib-Mb2 structure using two molecules each in the asymmetric unit. The structures were iteratively refined using refmac and phenix.refine (Version1.19.1) (65) followed by manual model building in COOT (66). Models were validated with MolProbity (67). Molecular structures were represented and rendered with ChimeraX (68, 69).

Crystals of Abl_64–510_ in complex with SKI and asciminib were obtained by combining 0.3 µL of 600 µM Abl_64–510_ + 700 µM SKI + 700 µM asciminib (~32 mg/mL) in 5% DMSO with 0.4 µL reservoir of 0.1 M Tris–HCl pH 8 + 1.75 M Ammonium sulfate + 2% (v/v) polypropylene glycol 400 (PPG 400). The final stock of complex was concentrated from 1 µM Abl_64–510_ with ~1.2 µM SKI/asciminib after incubation at 4 °C for 6 h. Screening around this condition yielded crystals in a transparent diamond-shaped or plate-shaped crystals. Crystals were grown at 18 °C by sitting drop for a few days. The crystals were transferred to a drop of fresh reservoir containing 20% xylitol with matching concentration of inhibitors in 5% DMSO for few seconds for cryoprotection.

Single crystal X-ray diffraction data were collected at 100 K at Advanced Light Source Berkeley (BL201). Data were integrated with XDS (61) as well as scaled and merged with AIMLESS (62). Analysis of processed data with phenix.xtriage (70) found outliers in the dataset and further revealed substantial translational noncrystallographic symmetry with a Patterson peak of 56.63% height relative to origin, complicating refinement. Initial phases were obtained by molecular replacement (PHASER) (64) using Abl-nilotinib-asciminib (PDB-ID: 5MO4) as a search model with two molecules in the asymmetric unit. The kinase domain, SH3, and SH2 (regulatory domains) were individually placed during molecular replacement. Refinement and manual model building were performed by phenix.refine (version 1.19.1) and Coot, respectively (65, 66). Models were validated with MolProbity (67). Molecular structures were represented and rendered with ChimeraX (68, 69) and PyMol (71).

## Supplementary Material

Appendix 01 (PDF)

## Data Availability

Structure factors and refined coordinates obtained from X-ray crystallography have been deposited into the Protein Data Bank (www.wwpdb.org) under PDB accession codes: 8SSP (72) (AurA-danusertib-Mb1), 8SSO (73) (AurA-danusertib-Mb2), and 8SSN (74) (Abl_64–510_-SKI-asciminib).
